# Myocardial fat quantification using navigator 1H MRS combined with MRI: myocardial fat fraction is inversely correlated with indexed left ventricular mass in healthy subjects

**DOI:** 10.1186/1532-429X-11-S1-P150

**Published:** 2009-01-28

**Authors:** Alban Redheuil, Ronald Ouwerkerk, Chia-Ying Liu, Joao AC Lima, David A Bluemke

**Affiliations:** 1 Johns Hopkins University, Baltimore, MD USA; 2 NIH, Bethesda, MD USA

**Keywords:** Leave Ventricular Mass, Index Leave Ventricular Mass, Water Suppression, Short Axis Cine, Left Ventricular Parameter

## Introduction

Myocardial fat content has recently been shown to be a potential indicator of myocardial involvement in diabetes or obesity. Myocardial triglycerides (TG) can be quantified non-invasively in humans with proton magnetic resonance spectroscopy (1H MRS). In this study, we quantified myocardial TG with an ECG and navigator gated 1H MRS sequence in combination with a cardiac MRI measuring left ventricular (LV) volumes, mass and function. Our aim was to study the feasibility of this protocol and the relationship of myocardial fat to LV mass in healthy subjects.

## Methods

All studies were performed on a 3.0 T MR scanner (Trio Tim, Siemens) on 20 healthy subjects (12 men, mean age: 42 ± 18 years), Myocardial 1H MRS spectra were obtained with an average 6-ml voxel positioned in the interventricular septum prescribed on two chamber, four chamber and short-axis images of the LV. Two MRS spectra were recorded with ECG gated PRESS, TR/TE = 1 R-R/30 ms, with navigator across the liver-lung interface to reduce of breathing effects. One spectrum (32 to 128 averages) was recorded with WET water suppression. Another spectrum (4 averages) was recorded without water suppression. Fat content was quantified with Amares/MRUI and related to water in unsuppressed spectra. LV volumes and mass were measured on short axis cine MRI images using QMass (MEDIS). The correlation between left ventricular parameters and myocardial fat fraction were studied using Pearson's coefficients.

## Results

**Figure 1:**
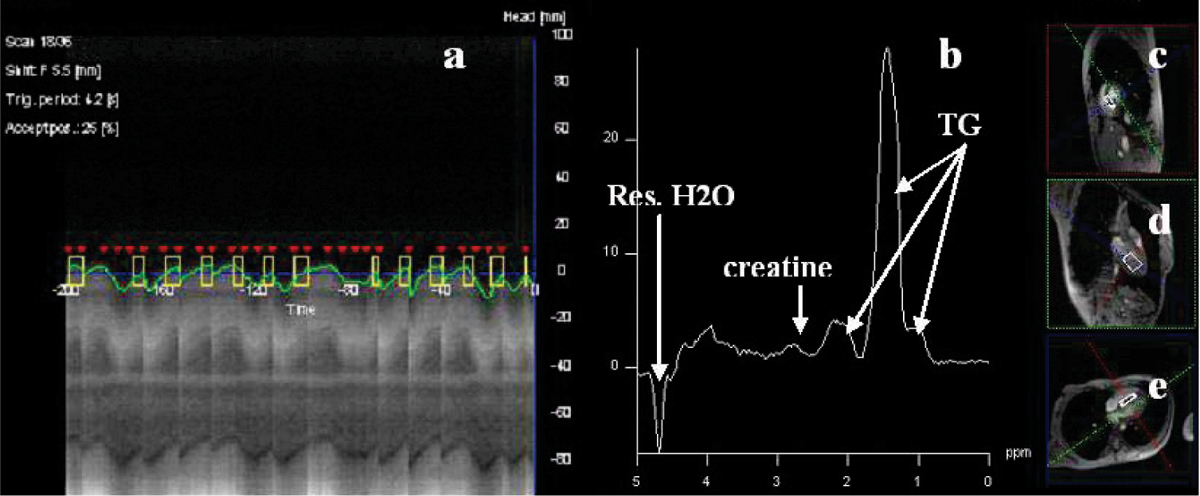
**Navigator signal (a) and water suppressed single voxel PRESS spectrum (b) with the selected volume superimposed on short axis (c), 2 chamber (d) and 4 chamber (e) 1H MR images from cine MRI**.

Figure 1 shows a water suppressed single voxel PRESS spectrum. The fat peak is clearly visible, but the creatine peak is relatively weak. In this subject fat/water ratio was found to be 0.8%. Shimming was of variable quality because this step could not be performed with both navigator and ECG gating. Nevertheless a fat/water ratio could be determined in all subjects. The fat/water ratio was 0.89 ± .9% (mean ± SD, n = 20 range 0.02 to 3.5). Mean ejection fraction was 67.5 ± 5.3%, mean systolic blood pressure:125 mmHg, mean body mass index: 28, mean body surface area (BSA): 1.87 m^2^ and mean LV mass was 129 ± 5 g. We found a negative correlation between LV mass and myocardial fat/water ratio (r = -0.51, p = 0.05) and a significant correlation between LV mass indexed by BSA and myocardial fat fraction (r = -0.56, p = 0.03).

## Conclusion

Myocardial fat fraction quantification using ECG and navigator gated 1H MRS was feasible within a comprehensive exam in healthy subjects and correlated significantly with indexed LV mass.

